# Quantitative Proteomic Analysis Reveals Novel Insights into Intracellular Silicate Stress-Responsive Mechanisms in the Diatom *Skeletonema dohrnii*

**DOI:** 10.3390/ijms20102540

**Published:** 2019-05-23

**Authors:** Satheeswaran Thangaraj, Xiaomei Shang, Jun Sun, Haijiao Liu

**Affiliations:** 1 Tianjin Key Laboratory of Marine Resources and Chemistry, Tianjin University of Science and Technology, No 29, 13th Avenue, TEDA, Tianjin 300457, China; satheeswaran1990@gmail.com (S.T.); shangxiaomei1987@126.com (X.S.); coccolith@126.com (H.L.); 2 Research Center for Indian Ocean Ecosystem, Tianjin University of Science and Technology, No 29, 13th Avenue, TEDA, Tianjin 300457, China; 3 Faculty of Food Engineering and Biotechnology, Tianjin University of Science and Technology, No 29, 13th Avenue, TEDA, Tianjin 300457, China; 4 Institute of Marine Science and Technology, Shandong University, No 27, Shanda Nan Road, Jinan 250110, China

**Keywords:** abiotic stress, silicate limitation, diatom, iTRAQ, proteomics, photosynthesis, carbon fixation

## Abstract

Diatoms are a successful group of marine phytoplankton that often thrives under adverse environmental stress conditions. Members of the *Skeletonema* genus are ecologically important which may subsist during silicate stress and form a dense bloom following higher silicate concentration. However, our understanding of diatoms’ underlying molecular mechanism involved in these intracellular silicate stress-responses are limited. Here an iTRAQ-based proteomic method was coupled with multiple physiological techniques to explore distinct cellular responses associated with oxidative stress in the diatom *Skeletonema dohrnii* to the silicate limitation. In total, 1768 proteins were detected; 594 proteins were identified as differentially expressed (greater than a two-fold change; *p* < 0.05). In Si-limited cells, downregulated proteins were mainly related to photosynthesis metabolism, light-harvesting complex, and oxidative phosphorylation, corresponding to inducing oxidative stress, and ROS accumulation. None of these responses were identified in Si-limited cells; in comparing with other literature, Si-stress cells showed that ATP-limited diatoms are unable to rely on photosynthesis, which will break down and reshuffle carbon metabolism to compensate for photosynthetic carbon fixation losses. Our findings have a good correlation with earlier reports and provides a new molecular level insight into the systematic intracellular responses employed by diatoms in response to silicate stress in the marine environment.

## 1. Introduction

Diatoms are a major group of phytoplankton, which play a significant role in the global carbon cycle [1], often thriving under adverse marine environmental conditions. It is known that diatoms can subsist during long periods of nutrient limitation [2] and form blooms following higher nutrient concentration [3]. Diatoms require a large quantity of silicon for their unique siliceous cell wall and cellular process [4]. It was reported that silicate deficiency led to diatom cell cycle arrest, which was reinitiated once silicate was provided [5,6].

In general, the response of nutrient limitations on phytoplankton photosynthetic mechanism is poorly understood [7], due to their cellular response variance to different nutrient stress [8]. A recent proteomic investigation on the diatom *Thalassiosira pseudonana* in response to iron and nitrate limitation showed PSII and PSI proteins were downregulated and, therefore, reduced its oxidoreductase activity [9,10,11]. Despite this, the diatom photosynthetic mechanism in response to silicate deprivation remains largely unknown. The response of nutrient limitations on diatom carbon metabolism has been widely discussed, i.e., nitrogen starvation [9], phosphorus limitation [12], silicon limitation [13], and iron limitation [11], however, intracellular mechanisms interlinked with electron transport, carbon metabolism, and ROS accumulation remains to be explored. 

There have been few studies on diatoms’ reactions with the oxidative response during nitrogen and phosphorus limitation [14] and iron starvation [15], while silicate limitation in these intracellular processes unknown. To date studies have mostly focused silicate limitation on diatom fundamental molecular mechanisms: cell cycle arrest [6], cell wall formation [4], carbon adjustment [13], and morphological changes [16]. Therefore, the mechanism underlying intracellular silicate stress-responses associated with oxidative stress, and ROS production are largely unknown.

Nutrient limitation on diatoms can produce reactive oxygen species (ROS) and induce programmed cell death (PCD) [17,18]. The preliminary characteristics of PCD have been described on diatoms *Thalassiosira weissflogii* under N stress or exposure to exogenous aldehyde [17,19]; *Ditylum brighwellii* under N and P stress [20]; *T. pseudonana* under Fe limitation [15]; and *Phaeodactylum tricornutum* under N stress [3], however, the mechanism underlying the association of PCD in diatoms intracellular silicate limitation remains elusive. In general, the cellular response of diatoms has some common characteristics to a variant stress condition, however, the ROS and PCD have been the exception of these due to their differential attribution to variant nutrient limitation [21]. In this manner, it is essential to investigate the possible activation of ROS and PCD instigated by Si-stress to understand the intracellular silicate stress-response on diatom. 

New introduction methods of isobaric tags for relative and absolute quantitation (iTRAQ) is a well-suited method to examine the distinct proteomic changes in cells acclimated to a different environmental condition. Here an iTRAQ-LC-MS/MS proteomic approach was used to examine the comparative proteomic investigation in *Skeletonema dohrnii* under silicate deplete and replete condition, respectively. The purpose of this comparative study is to understand the different metabolic regulation involved in the intracellular silicate stress-response especially those associated mechanism controlling cell growth, replication, carbon fixation, and inducing oxidative stress and possible ROS production. The findings revealed underlying molecular mechanisms controlling cell growth, replication, and photosynthetic carbon fixation under depleted silicate conditions in the marine environment. 

## 2. Results

### 2.1. Physiological Responses of the Cell

**Figure 1:**
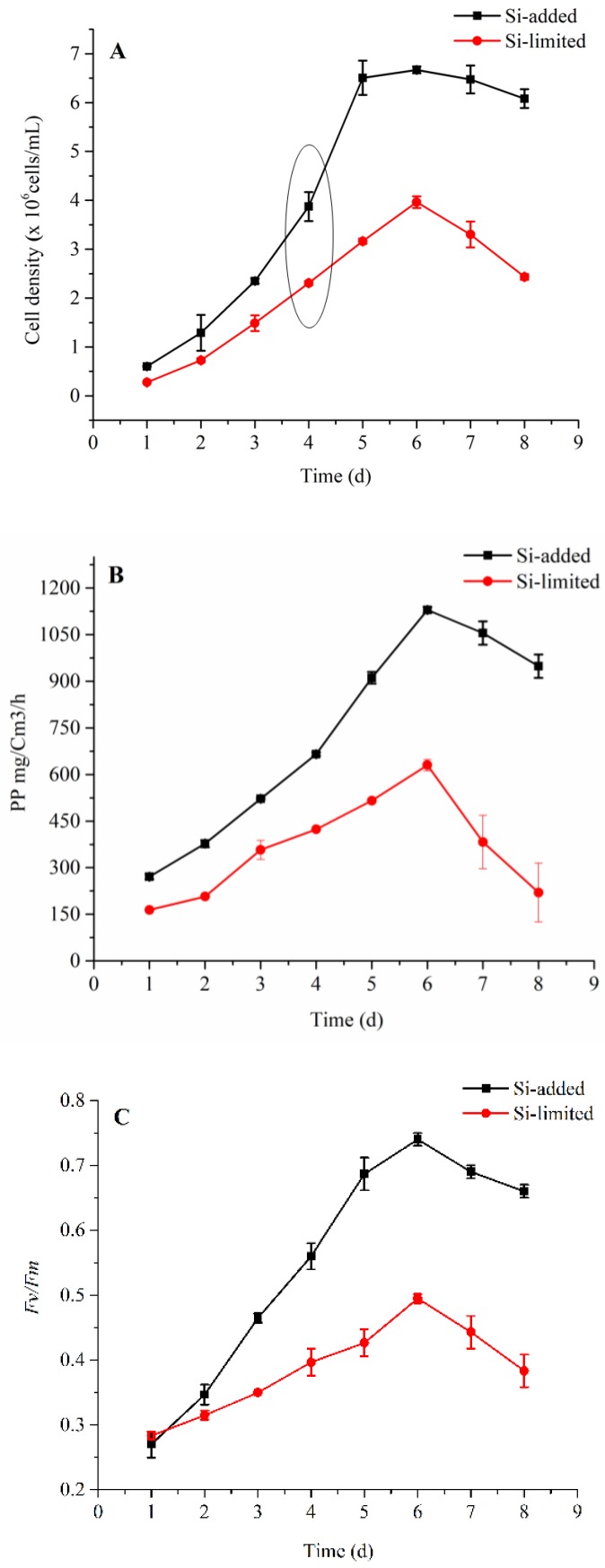
Cell density, net primary productivity, and photosynthetic efficiency in Si-limited and Si-added samples of *S. dohrnii.* Cell density (**A**), net primary productivity (**B**) and photochemical quantum yield of PS II (*FV/FM*) (**C**). The circle in Figure A shows the duration of sample collection for iTRAQ proteomics. The error bars represent the standard errors from triplicate measurements.

Physiological changes of *S. dohrnii* under Si-deplete and Si-replete conditions are shown in (Figure 1). Cells subject to Si restriction grew slower all through the experiment than the Si-included sample (Figure 1A). Furthermore, the cells were under Si-constrained declined quickly once they achieve a higher density, while Si-included cells declined gradually. The net primary productivity NPP showed a significant variation between the treatments, particularly in the expositional stage (day 5) where cells exposed to silicate supplementation NPP expanded radically, whereas, in the Si-limited group it was 50% slower than the Si-included example (Figure 1B). The photochemical effectiveness of PSII, *Fv/Fm* in the expositional stage notably increased in Si-added condition, while in Si-restricted cells it increased gradually (Figure 1C). Together these cellular performance measurements indicate the strong dependence of the *S. dohrnii* integrated physiology on Si-bioavailability. 

### 2.2. iTRAQ Results

To unravel *S. dohrnii* intracellular responses to Si-stress, we harvested cells for iTRAQ based proteomic analysis on day 4. Four biological replicates of the samples have been labeled and mixed for proteomic profiling. In whole 364,183 spectra, 3892 peptides, and 1768 proteins were identified with 1% of FDR. Detailed information including accession numbers, peptide sequences, protein description, number of spectra, *p*-value and *q*-value and repeatability test between the samples are given in (Supplementary Table S1). Of these identified proteins using a significance cut-off of a two-fold change and a *p*-value less than 0.05, we have identified 594 differentially expressed proteins between the Si-restricted and Si-added samples, of which 345 proteins were downregulated (Supplementary Table S2), and 249 were up-regulated (Supplementary Table S3), respectively. 

### 2.3. Gene Ontology and COG Analysis

**Figure 2:**
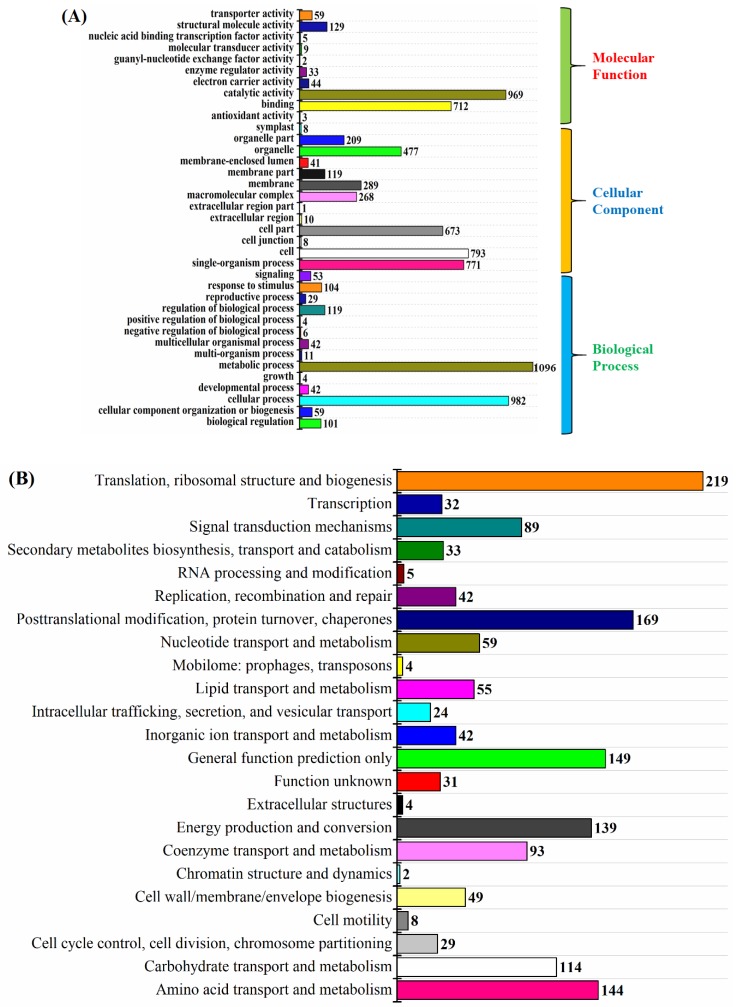
(**A**) Bar plot of Gene Ontology analysis; the different color shows different GO category. (**B**) The diagram shows the bar plot of COG analysis *x*-axis displays the COG term; *y*-axis displays the corresponding protein count illustrating the protein number of different functions.

To additionally explore the potential role of overall identified proteins we used GO terms, which comprised three sets of ontologies: molecular functions (MF), cellular components (CC), and biological process (BP) (Figure 2A). As shown in (Figure 2A) the most represented GO terms in the MF category was a catalytic activity, and protein binding consisting of electron transport-transferring electrons within the cycling electron transport of photosynthesis activity. In the CC category, the cellular process and cell cyclic, including photosystem II, photosystem I, chloroplast thylakoid membrane, and proton transport proteins were significantly enriched. For the BP category biological regulation, including protein-chromophore linkage, regulation in ATP synthesis coupled proton transport, regulation in photosynthesis, and glycolysis, were the most important enriched term. Furthermore, the overall classified proteins were subjected to COG enrichment analysis to understand the cellular metabolism influenced by the Silicate deprivation. COG functional classification result shows that proteins were involved in almost every aspect of *S. dohrnii* metabolism (Figure 2B). However, among all groups, translations, ribosome structure, and biogenesis were the largest group (219) influenced by lower silicate, followed by post-translational modifications, protein turnover, chaperones (169), and general function proteins (149). 

### 2.4. KEGG Analysis of the DEPs in Skeleteonema dohrnii

**Figure 3:**
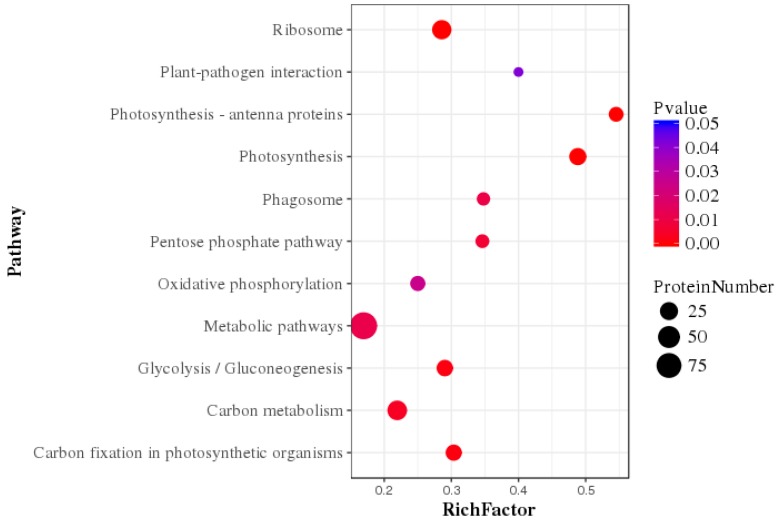
Statistics of pathway enrichment factor analysis of differentially expressed proteins comparing of Si-limited vs. Si-added conditions.

The Kyoto Encyclopedia of Genes and Genomes (KEGG) enrichment analysis was performed to determine potential clustering of the differentially expressed protein in a specific metabolic pathway. Using the KEGG database 594 DEPs were annotated and classified into 91 metabolic pathways. The main KEGG pathway classification of DEPs are photosynthesis (*p* < 0.01), photosynthesis antenna (*p* < 0.01), and oxidative phosphorylation (*p* < 0.01), glycolysis/gluconeogenesis (*p* < 0.03), carbon fixation in photosynthesis organism (*p* < 0.01) and pentose phosphate (*p* < 0.02) (Figure 3). 

### 2.5. Photosynthesis Metabolism

The multi-subunit protein complex of PSII is embedded in the thylakoid membrane of algae that drives a water-plastoquinone oxido-reductase. The overall differentially abundant proteins in photosynthesis metabolism of this study are given in (Supplementary Table S4). Result shows that Si-limitation caused decreased abundance of seven primary proteins in the PSII complex, i.e., D1 (PabA, −1.52-fold), D2 (PsbD, −1.44-fold), CP43 (PsbC, −1.51-fold), CP47 (PsbB, −1.26-fold), cytochrome b559 subunits (PsbE, −1.64-fold; PsbF, −1.43-fold; and PsbQ, −0.12-fold). Decreased abundance of these proteins influenced in cytochrome b6*/f* complex; resulting in two primary proteins being decreased in abundance (PetD, −1.12-fold; PetA, −1.36-fold). The impact in PSII and cytochrome b6*/f* further downregulated six proteins in PSI, i.e., (PsaA, −1.35-fold; PsaB, −1.68-fold; PsaC, −0.63-fold; PsaF, −0.83-fold; PsaJ, −0.92-fold, and PsaL, −2.21-fold) which could damage the electron transfer from PSI to NADP [22]. 

Fucoxanthin and chlorophyll are known as major proteins in the LHCs of diatoms. In the present study 13 pigment proteins were downregulated, consisting of five FCPs and five Chl proteins and three accessory proteins (Supplementary Table S4), which may influence in the process of exciting energy and chlorophyll biosynthesis. In addition, 26 functional proteins associated with ATP metabolism were differentially expressed, of which 16 proteins were downregulated and 10 proteins were upregulated (Supplementary Table S4). Functional proteins associated with quinone binding, ATP synthase, proton transport, and rotational mechanism were notable down-regulated proteins, whereas mitochondria, vascular, and sulfur proteins were noteworthy up-regulated proteins. 

### 2.6. Carbon Metabolism

The state transition of photosynthesis metabolism due to silicate deprivation was expected to impact on carbon metabolism. Nevertheless, results showed an increased abundance of carbon metabolic proteins rather than decreasing. Nine proteins associated with glycolysis were differentially expressed and, among them, two were downregulated and seven were up-regulated (Supplementary Table S4). The up-regulated proteins of (fructose1,6-biphosphate; triose phosphate isomerase; glyceraldehyde-3-phosphate dehydrogenase; nicotinate-nucleotide pyrophosphorylase; phosphoglycerate kinase; and enolase) involved in the glycolytic process of step 1 (hexokinase), step 2 (phosphoglucose Isomerase) and step 3 (phosphofructokinase). As these upregulated proteins could enhance the production of pyruvate, six functional proteins were upregulated in pyruvate metabolism in this study. Upregulated proteins of (phosphoenolpyruvate; D-2 hydroxy-acid dehydrogenase; pyruvate dehydrogenase; pyruvate kinase; pyridine nucleotide transhydrogenase; acetyl-coenzyme) would enter mitochondria to produce GTP and FADH2. In addition, many functional proteins in TCA metabolism were upregulated, i.e., (isocitrate; oxoglutarate, succinyl; succinate; malate; dihydrolipoamide acetyltransferase; dihydrolipoyl dehydrogenase; pyruvate carboxylase; fumarate hydratase; and pyridine nucleotide), showing the interlinking pathway of diatoms’ central carbon metabolism under silicate stress.

### 2.7. Response of Cellular Respiration

The respiratory chain has protein complexes of cytochrome I, II, III, IV, that transfer electrons from NADH to O2 via redox. The present result shows downregulation of two NADH-ubiquinone reductases (XP_002292597.1, −1.37-fold; AAZ99426.1, −1.32-fold), and cytochrome complex I (YP_316586.1, −1.49-fold), cytochrome *c* oxidase complex II (AOE43471.1), and cytochrome *c* oxidase subunit 6B1, complex IV (EED96133.1, −1.74-fold) (Supplementary Table S2), shows an influence in electron deliver. Further downregulation of mitochondrial alternative oxidase (AOX) (XP_002294653.1) and many FoF1-type ATP synthase proteins (Supplementary Table S4) indicates the possible impact on ATP production. 

### 2.8. Identification PCD Related Proteins

It is known that superoxide dismutase and peroxiredoxin enzymes replace ROS scavenging function, however, in the present study, both proteins were down-regulated (superoxide dismutase, EJK75419.1; peroxiredoxin EED94096.1) which may impact the defense mechanism against ROS. In general, heat shock proteins protect the cellular mechanism when cells exposed to a stressful condition. Nevertheless, in this investigation downregulation of chaperonin GroES, EJK46404.1; molecular chaperone, XP_002286821.1; and some co-expression proteins like FKBP-peptidyl-prolyl cis-trans isomerase, EJK59733.1; and trypsin-serine proteases, EJK75980.1 may increase the impact of the molecular mechanism by Si stress. Additionally, downregulation of an anti-apoptotic protein of chaperone GroEL (HSP60 family, ACI64273.1) was observed in Si-limited cells. Furthermore, upregulation of RuBisCO large subunit (ABF60361.1) and downregulation of RuBisCO small subunit (BAA75795.1) may lead cells to oxidative stress under Si-limited conditions.

Moreover, in comparing with silicate-deplete and -replete cells, four nutrient transporters were differentially abundant. Among them, nitrate/nitrite transporter (NRT2) (EJK46860.1) was downregulated, whereas 5′-nucleotidase, (XP_002295180.1), sulfate adenylyltransferase, (XP_002286746.1), and sulfite reductase, (EED93364.1) were upregulated. 

Additionally, photosynthesis and carbon metabolic regulation, results showed that silicate stress further regulated numerous cellular metabolism of *S. dohrnii*. To unravel the whole impact, an iPath global metabolic pathway was annotated (Supplementary Figure S1). The iPath biochemistry map shows Si-stress also regulated amino acid, nucleotide, and lipid metabolisms. Many proteins involved in the biosynthesis of purine and pyrimidine were down-regulated (Supplementary Table S4), which were associated with amino acid metabolisms, i.e., DNA binding (EJK63460.1), RNA polymerase (YP_009093166.1), adeno succinate synthase (XP_002292359.1), and de novo IMP biosynthetic process (EJK50540.1).

## 3. Discussion

The goal of this study was to better understand the marine diatom *S. dohrnii* metabolic responses when exposed to a silicate-limited environment by examining the expressed proteome at the exponential growth stage. However, why proteomic? Why not genomic or transcriptomic? Few investigators have already reported gene expression of diatoms to Si-limitation [13,23]. Furthermore, recent functional RNA study showed that although much of the genome was transcribed, not all the transcriptions were translated into functional proteins [24,25]. This suggests that genomic and transcriptomic studies may be limited to understand the cellular metabolomic changes to the changing environment deeply. Similarly, diatoms change their transcript expression into a small range of protein expression under nutrient limitation suggesting diatoms, like other organisms, may rapidly increase the transcript abundances, but not all translate into proteins [26]. Therefore, an iTRAQ quantitative proteomic approach was applied to *S. dohrnii* to understand accurately the underlying intracellular mechanisms in response to silicate stress. 

### 3.1. Physiological Changes

The growth rate of *S. dohrnii* in the present study varied based on the silicate concentrations of which could be the reason for silicate demand to their cellular processes. Previous reports noted that Si-stress induced the aging early of diatoms, therefore, regulated metabolism inside and reduced cell growth rate and replication [6,13,27]. These reports also support the observed variation of NPP between Si-limited and Si-added samples. A further difference in *Fv/Fm* shows the inefficient activity of PSII complex, could be the attribution of LHCs responses to the Si-concentration [28,29]. The present study GO and COG results (Figure 2B) showed the similar detection of proteins on diatom *T. pseudonana* under silicon limitation, [4] indicates diatom’s common metabolomic regulation under silicate stress. 

### 3.2. Downregulation of Photosynthesis

**Figure 4:**
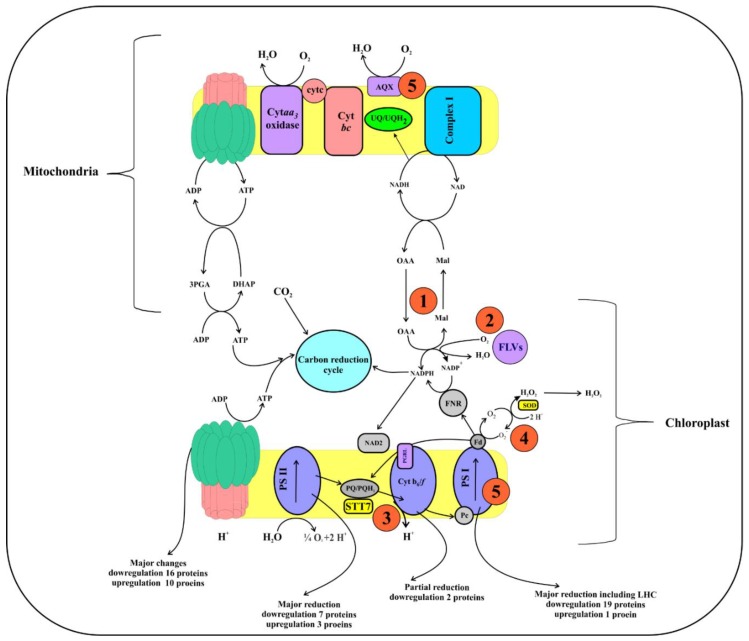
A schematic diagram of the electron transfer pathway in oxygenic photosynthesis. During the reaction, the reducing equivalents generated at PSII and transferred to plastoquinone then cytochrome, plastocyanin, PSI, Fd, and ferredoxin NADP+. Electron transfer/flow around PSI is mediated by PGRL1 and NAD2. Reduction in the electron flow around PSII, cytochrome, and PSI resulting in a deficiency in ATP, which is compensated by different mechanisms depending on the demand for ATP. For example, (1) A mitochondrial respiration-based first compensating mechanism likely involving malate/oxaloacetate shuttle, that reduced energy from the chloroplasts to cytosol, and eventually mitochondria. After that, the reducing energy in the mitochondria are converted as ATP by electron transport, which would return to the chloroplast by the ATP translocator (2), the probable occurrence of oxygen photoreduction at the level of NADPH with the help of FLV proteins, which allows ATP biosynthesis via pseudo-cyclic phosphorylation (3). When mechanism (1) and (3) are highly activated, the plastoquinone becomes more reduced, resulting in STT7 kinase activation, therefore, altering LHC phosphorylation and state transition (4). The higher decrease in PSI acceptors would induce the Mehler reaction by direct O_2_ photoreduction through reduced Fd, therefore making H_2_O_2_ (5), and ROS accumulation then induces the AOX and affects the PSI protein complex and decreases the PSII/PSI ratio. Furthermore, downregulation of PSII, cytochrome, and PSI proteins show it makes fewer reducing equivalents flow across a chloroplast and mitochondria, thus resulting in lower ATP production and impact on PSII and PSI, and the LHCs of *S. dohrnii*.

PSII in plants referred to as an engine of earth and this complex affected first when plants expose to any abiotic stress [30]. Decreased abundance of PSII proteins in this study showed a reduction in catalyzing electron production, and transport, therefore, only limited electrons were pumped across a chloroplast. The schematic diagram (Figure 4) describes pathways of electron flow between chloroplast and mitochondria during oxygenic photosynthesis; also it shows five compensating mechanisms that rectifying process during the reduction in electron flow. Fascinatingly, results showed that, besides the reduction of electron production and transfer, Si-stress also influenced in the four compensating mechanisms of electron transfer, i.e., down-regulated malate carrier protein in mitochondria (XP_002295632.1, −1.4-fold) shows a reduction in first compensating mechanism [23], while serine/threonine-protein kinase (STT7kinase) downregulation (XP_002288169.1, −1.17-fold) displays a reduction in the third step and alteration in LHCs [31]. Furthermore, the decreased abundance of ferredoxin (EJK54785.1, −0.9-fold), and AOX protein (XP_002294653.1, −1.74-fold) indicates the failure of the fourth and fifth compensating mechanisms [32]. These results show that during silicate limitation of *S. dohrnii* cells the electron flow between chloroplasts and mitochondria were altered. 

Regulation in PSII electron flow would damage the PSI and LHCs [33]; therefore, it is not surprising 13 proteins in the pigment metabolism were decreased in abundance due to Si-stress. Similarly, in diatom *T. pseudonana* six chlorophyll biosynthesis genes were downregulated due to silicon stress [13]. Chlorophyll is a nitrogenous compound; any impact on these proteins would decrease the nitrogen demand of the cell and diminishes their light-capturing efficiency, therefore, inducing oxidative stress and ROS production [9]. Downregulation of these proteins also affects the initial process in energy production, and carbon fixation [34], shows silicate is a vital nutrient for diatom carbon fixation to facilitate the proper electron transport and catalyze the reaction. Differential proteins abundance in this study because of Si-limitation reduced electron flow and increasing the chances of oxidative stress and ROS (Figure 4). Reduced silicate assimilation in diatoms caused the changes in protein abundance, resulting in metabolic imbalances to the oxidative stress [26]. Up-regulation of metabolomic sulfur protein in this study could also be linked to oxidative stress because it has a role as a compatible solute, is thought to be a part of the cellular antioxidant [35], and its synthesis and excretion might dissipate the amount of excess energy, carbon, and reducing the equivalents under nutrient starvation [36]. Of which indicates reducing demand for carbon skeletons to the central carbon metabolism under silicate stress conditions. 

In total, our findings show *S. dohrnii* under Si-limitation retain PSII, cytochrome, and PSI complexes, however, major functional proteins of these subunits were down-regulated. Hence, failure to produce enough electrons and protons to pump across the thylakoid membrane (Figure 4); therefore, a regulation in antenna complex that limits the initial step of carbon storage. Furthermore, the inadequate flow of electrons and protons also affected ATP metabolism to drive ATP synthase, therefore, resulting in less ATP synthase complex during the silicate stress that controls *S. dohrnii* cell growth, replication, and energy storage for other metabolic functions depending on these energies. 

### 3.3. Reduction in the Photosynthetic Carbon Fixation

**Figure 5:**
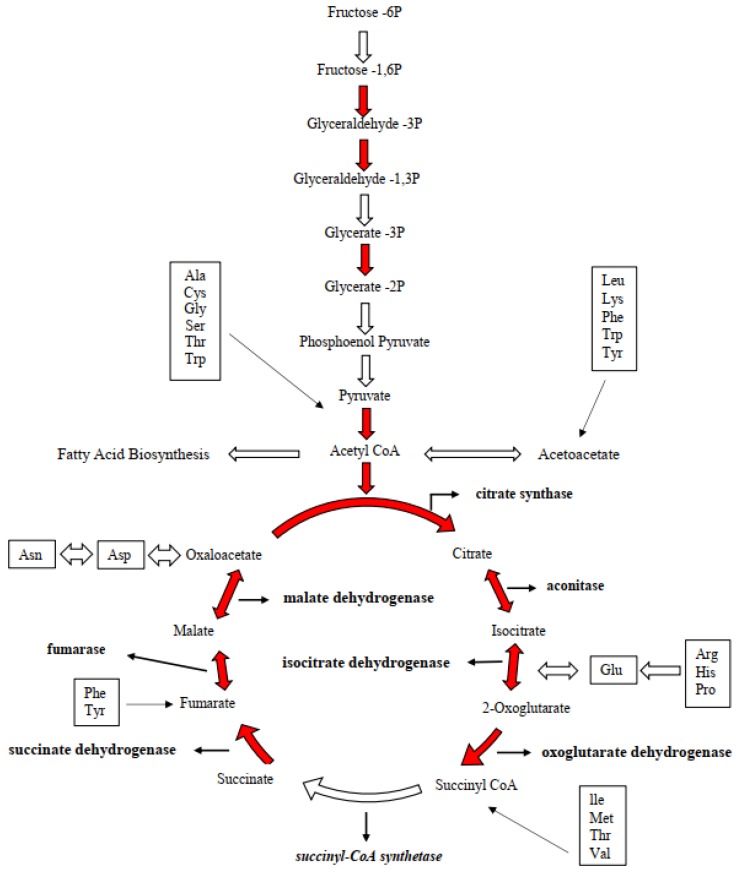
Representation of the changes in proteins abundance associated with carbon metabolism in *S. dohrnii* at the onset of silicate limitation. Increases are shown in red arrows. Boxes show where carbon skeletons from amino acid feed into the pathway.

The upregulation of glycolic activity lead-carbon from intracellular stores to the central carbon metabolism. However, the upregulation of proteins catalyzed in the reverse reaction of gluconeogenesis in the present study showed an enhancement in the pyruvate metabolism. Similar results were observed in the transcript level of diatom *T. pseudonana* [13]. Observed upregulated proteins in the carbon metabolism show the direction of carbon flow in *S. dohrnii* (Figure 5). Pyruvate is the product of glycolysis, which can be converted into acetyl-CoA, also shows a unidirectional supporting of increasing glycolytic activity. Acetyl-CoA not only used for the carbon input to the TCA as a source of energy and reducing equivalents, but it also can be used for the fatty acid biosynthesis process, which provides a carbon skeleton for the silicate assimilation and biosynthesis compounds of fatty acids (Figure 5). Up-regulated proteins in the TCA cycle of this study shows carbon from glycolysis flowing through acetyl-CoA and then into the TCA cycle. All these findings reveal *S. dohrnii* cells in Si-stress reduced silicate assimilation; the demand of energy reducing equivalent, and carbon skeletons, in response to this photosynthetic carbon fixation was decreased.

If carbon fixation was decreased because of the less demanding carbon skeleton and reducing equivalent then why were the glycolysis, pyruvate, and TCA metabolic proteins upregulated? Interestingly, similar results were seen on cyanobacterium under nitrate limitation [22], diatoms under the iron [11], and nitrogen limitation [9]. These authors propose that, during nutrient limitation, cells’ adaptation to the subsistence and the breakdown of intracellular stores is a more efficient source of carbon for the reassimilation of the nutrient than photosynthesis. These findings also show that the impact of Si-limitation in diatom carbon metabolism has a similar role as iron and nitrate. 

Diatoms have a complete urea cycle [1], therefore, demanding higher carbon skeletons and nitrogen molecules than available in the organisms [37]. In the present study, reduction in the nitrogen molecule from chlorophyll pigments and carbon skeletons by Si-limitation regulated the *S. dohrnii* urea cycle (Supplementary Figure S1). Moreover, observed *S. dohrnii* carbon metabolism suggested up-regulation of acetyl-CoA was a forerunner for fatty acid biosynthesis (Figure 5). This statement provides a question to explain if acetyl-CoA is diverted into fatty acid biosynthesis what would be the origin for the TCA cycle? Notably, most of the TCA cycle processes are not only unidirectional, but it catalyzes the reverse reactions in a specific circumstance [9]. The TCA cycle processes are adjustable and expected to reflect the metabolomic and physiological processes as per the cell’s demands [38]. This finding shows the carbon metabolic process of *S. dohrnii* under Si-limitation react to various TCA intermediates that decide the carbon flow to the entire central carbon metabolism than to be directed and altered to fatty acid metabolism. In total, reduction in carbon skeletons and nitrogen compound molecules by Si-limitation regulated central carbon and fatty acid metabolisms of *S. dohrnii* under silicate stress.

### 3.4. The Response of Cellular Respiration to Possible ROS Accumulation

**Figure 6:**
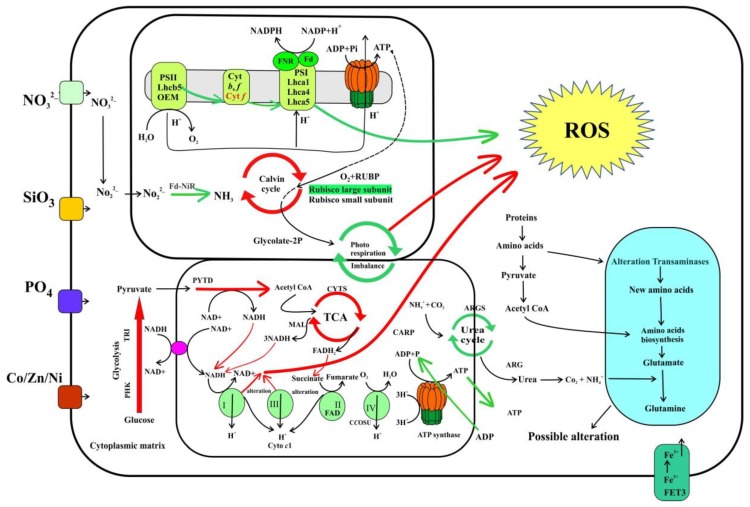
Identified energy and carbon metabolomic pathways and processes of *S. dohrnii* under Si-limited-Si-added condition. All red arrows indicate up-regulated proteins and processes and green shows downregulation proteins and processes. PS: photosystem, Lhc: light-harvesting complex, OEM: oxygen-evolving complex, Cyt: cytochrome, Fd: ferredoxin; FNR: ferredoxin-NADP+, Fd-NiR: ferredoxin nitrite reductase; TCA: tricarboxylic acid, RUBO: RuBisCO; ROS: reactive oxygen species, PHK: phosphoglycerate kinase; TRI: triosephosphate isomerase; PYTD: pyruvate dehydrogenase; CYTS: citrate synthase; MAL: malate/lactate dehydrogenase; CARP: carbamoyl-phosphate synthase; ARGS: argininosuccinate synthase; ARG: arginase, complex I; NADH ubiquinone reductase, complex III; cytochrome *c*1.

A schematic diagram (Figure 6) shows the identified energy and carbon metabolic pathways in this study and their metabolic regulation by Si-limitation to possible ROS production. Complexes I and III are the primary sites for electron transport and ROS generation [39]. In the present study downregulation of NADH-ubiquinone reductases and complex I suggested a possible enhancement of ROS from cytochrome complex I. Earlier investigations described that oxidation of either complex I or II substrates when complex III is altered and increased ROS accumulation [39,40]. Though cytochrome *c* oxidase is not a direct source of ROS [41], cytochrome *c* oxidase subunit 6B1 downregulation would facilitate the chances of ROS production from complexes I and III [42]. Therefore, it is proposed that the downregulation of cytochrome *c* oxidase subunit 6B1 in this study may inhibit complexes I and III and block the electron delivery to induce the ROS. Similar results were observed in transcriptome and proteome level in *T. pseudonana* at the onset of nitrate, phosphate, and iron limitation [14,15]. 

The mitochondrial alternative oxidase (AOX) protein used for removing an excess electron in the nutrient-limited diatom [43]; upregulation of this protein was involved in the mitigation of mitochondrial ROS production [14]. However, downregulation of this AOX protein and many essential enzymes of the F1 region of FoF1 type proteins (Supplementary Table S2) in this study suggests the reduction of ATP production with a coincided blockage of the respiratory chain and more possible chances of ROS production. Similar results were observed in the proteome level on diatom *T. pseudonana* in response to Fe limitation [15]. Although in the present study, direct observation of ROS production has not been identified, from the above findings in the proteome level, and comparing with similar findings on diatom it is predicted that due to inefficient electron transport in the photosynthetic and respiratory chain (Figure 6) leads to a possible ROS production under Si-limitation. These statements are consistent with the previously published reports [14,15] on diatoms in response to nutrient limitations.

### 3.5. Metabolic Regulation of PCD Related Proteins

The previous investigation noted that ROS caused by environmental stress can induce the PCD [44]; therefore, ROS cells must have a defense mechanism. However, in this study, some typical ROS defense proteins were decreased in abundance because of the Si constraint, such as superoxide dismutase, which has been earlier cloned and characterized in diatoms [42]; peroxidase I, which allows the enzymes to react with hydrogen peroxide [18]; peroxiredoxin, an antioxidant enzyme to detoxify peroxidase substrate [45]; and thiol-disulfide, an essential antioxidant protein that can combat oxidative stress by detoxifying [46]. Furthermore, the decreased abundance of chaperonin GroEL and other heat shock proteins shows the opportunity of increasing the impact on cytoplasm and apoptosis [47]. Photorespiration plays a vital role in excess energy dissipation under stress condition [48]. Along these lines, regulation of RuBisCO in this study shows an imbalance in photorespiration to alleviate oxidative stress. Interestingly, a serine protease was downregulated in this study, whereas adenine nucleotide translocator was upregulated, which would induce the caspase-specific activity and apoptosis [49,50]. Though the expression of these cell death-related domains may involve in the PCD of Si-limited *S. dohrnii* cells, the detailed mechanisms, i.e., biochemical role of this domain, detailed regulatory pathways in the *S. dohrnii* PCD require further investigation. 

Nitrate transporters (NRT2) in plants are responsible for nitrate uptake and deliver to various cellular parts; regulation of this transporter by abiotic stress affects the cellular process [51]. Recently, Hippler et al. [52] reported that Cu stress leads to downregulation of NRT2 and affect the cellular process of *Arabidopsis*. Likewise, in this study downregulation of NRT2 shows alteration in the nitrate transport leads to lower nitrate assimilation for diatom cellular process. A similar finding was reported on the *T. pseudonana* response to nitrate limitation [53]. 

Generally, purine and pyrimidine compounds are nitrogen originates from amino acids [54]. Changes in purine and pyrimidine could regulate amino acid metabolites and cell growth [55]. Therefore, it is assumed that the downregulation of 14 proteins caused the impact of amino acid binding and further associated metabolic activity of *S. dohrnii.* These included multiple aminotransferases that can yield many fates for amino acids: intracellular recycling to alpha-keto acids, pyruvate, or ammonia, and re-organization into new amino acids and completing the intracellular activity of the cell [56]. The whole impact of Si constraints on *S. dohrnii* metabolism are shown in Supplementary Figure S1, indicating the dynamic role of silicate stress on intracellular diatom metabolism. Our findings are in line with previous transcriptomic and proteomic studies of diatoms and other phytoplankton in response to the nutrient limitation [4,9,11,12,14,15].

## 4. Materials and Methods

### 4.1. Species Description

The cylindrical type of marine centric diatom *Skeletonema dohrnii* was first discovered by Sarno et al. [57]. In general, members of *Skeletonema* genus has been considered as a key species in diatoms research due to their global distribution, tropic importance to grazers and their similar physiology with other diatoms [58], therefore, we used *S. dohrnii* as a model species in this experiment.

### 4.2. Culture Condition

The marine diatom *Skeletonema dohrnii* was isolated from the Yellow Sea and cultured in the f/2 medium in the laboratory (25 °C 100–120 μmol photons m^−2^·s^−1^, with 14:10 light-dark cycle). Artificial seawater media (ASW) [59] was used with silicate stock solution (28.40 g·L^−1^) in this experiment, however, for the silicate-deplete and -replete conditions modified silicate concentrations (silicate limited 0.5 mL/L) and (silicate added 1.5 mL/L) were used in a controlled incubator (25 °C 150 μmol photons m^−2^·s^−1^) with a 14/10 h light/dark ratio using cool white fluorescent light. Both the cultures (Si-limited and Si-added) were grown separately in 1000 mL Nalgene and, during the experiment, pH was in a range of 7.5–8.0. The duration of the experimental phase was eight days. 

### 4.3. Analysis of Physiological Measurements

The cell numbers were counted by using an AE 2000 inverted microscope (Motic Group Co., Ltd., Xiamen, China) and Qiujing hemocytometer at the same time every day. The cell density was calculated as follows: CD = (*N*/80) × 400 × 104, where CD is the cell density and *N* is the cell abundance counted in 80 grids on the slide [60]. The photochemical quantum yield of photosystem (*Fv/Fm*) was measured by a Fast Ocean FRRF3 sensor with an Act2 system by the following procedure: Samples were initially kept in the low light to allow the oxidation of the electron transport chain (ETC) and relaxation of NPQ. The single-turnover protocol consisted of 100 flash lets (Fet, a single 1 µs excitation pulse from the LEDs within an FRRF3 sensor) with 2.0 µs Fet pitch (interval between the start of one Fet and the next) were set in the instrument. Then each sample was exposed sequentially to 12 actinic background irradiances spanning from 0–1200 µmol quanta m^−2^·s^−1^ to retrieve fluorescence light response curves. In the dark-adapted state, the maximum quantum efficiency of PSII was calculated using the ratio of *Fv/Fm* as per Wei et al. [61]. 

The ^14^C method was used for the net primary productivity (NPP) as follows: The inoculum was transferred to 30 mL incubation flasks in triplicates for the 24 h incubation. Before the incubation, 100 μL aliquots were transferred to a scintillation bottle from one sample and soaked in 10 mL scintillation cocktail. Three dark bottles were set up for control. The culture bottles were covered with different pore size meshes to create light gradients (0–800 PAR) after adding 20 μCi^14^C sodium bicarbonate. The incubation samples analyzed by Wallac System 1400 liquid scintillation counter (PerkinElmer Life and Analytical Sciences Inc., Wellesley, MA, USA) according to the manufacturer’s protocol.

### 4.4. Protein Extraction and Preparation

According to the cell cycle progression of diatoms, day 4 (mid-exponential growth) was chosen as the sampling time point for iTRAQ proteomic analysis. Protein extraction was carried out according to the description by Du et al. [4]. Briefly, one liter of culture from each sample (4 Si-deplete + 4 Si-replete = 8 samples = 8 L), collected through 2 μm pore-size filter membrane. Then the cells were resuspended with 10 mL medium into a 15 mL centrifuge tube. Once the pellets were collected by the centrifugation at 3000× *g* for 5 min, immediately 10 mL TRIzol Reagent added (Invitrogen, Life Technology, Carlsbad, CA, USA), and then the protein was extracted according to the manufacturer’s protocols. 

For the protein preparation, the protein pellets were resuspended in a lysis buffer (8 M Urea, 40 mM Tris-HCl, 2 mM EDTA, pH 8.5), then incubated with additional of 10 mM dithiothreitol (DTT) and placed at 56 °C for 1 h to reduce the disulfide bonds. After that 55 mM iodoacetamide (IAA) was added and samples were incubated with 45 min to block the cysteine residues proteins. The reduced protein mixtures were then precipitated by adding acetone at −20 °C overnight. After centrifugation at 4 °C, 25,000× *g*, for 20 min the pellet was dissolved in 0.5 M TEAB (triethylammonium bicarbonate, Applied Biosystems, Milan, Italy) and sonicated in ice. After centrifugation at 25,000× *g* at 4 °C, an aliquot was taken for the protein quantification by Bradford Assay according to the manufacturer’s protocol. The supernatants were kept −80 °C until further analysis. 

### 4.5. iTRAQ Labeling and Fraction

A total of 100 μg of proteins from each sample was digested with Trypsin Gold (Promega, Madison, WI, USA) with the ratio of protein/trypsin = 40:1, at 37 °C overnight. After trypsin digestion, peptides were dried with vacuum centrifugation then reconstituted in 0.5 M TEAB and proceeded based on the manufacturer’s protocol. In the present study, an experiment setting of 4:4 (8-plex, four biological replicates) was selected and labeled with different iTRAQ tags. The silicate replete samples were labeled with iTRAQ tags 113, 115, 117, 119, and silicate deplete samples were labeled with iTRAQ tags 114, 116, 118, and 121, respectively. The labeled peptides with isobaric tags were then incubated at room temperature for 2 h, and labeled peptides were then pooled and dried by vacuum centrifugation. 

The strong cationic exchange chromatography was performed using a Shimadzu LC-20AB HPLC equipped with a UV–VIS detector (Shimadzu, Kyoto, Japan). The fractionated peptides were first reconstituted with buffer A (5% ACN,95% H_2_O, adjust pH to 9.8 with ammonia) then loaded onto Gemini C18 5-μM, 4.6 × 250 mm reverse phase column containing 5-μm particles (Phenomen-ex). The peptides were separated at a flow rate of 1 mL/min with a gradient of 5% buffer B (5% H_2_O, 95% ACN, adjust pH to 9.8 with ammonia) for 10 min, 5–35% buffer B for 40 min, 35–95% buffer B for 1 min. The system is then maintained in 95% buffer B for 3 min before equilibrating buffer B for 10 min before the next injection. The elution was monitored by measuring absorbance at 214 nm, and fractions were collected at every 1 min. The eluted peptides were then pooled as 20 fractions, desalted with Strata X C18 column (Phenomenex) and vacuum-dried according to the manufacturer’s protocol. 

### 4.6. LC-MS/MS Analysis

Every fraction was then resuspended in buffer A (2% ACN, 0.1% FA in water) and centrifuged at 20,000× *g* for 10 min. The final concertation of the peptide was 0.5 μg/μL on average. The supernatant was then loaded onto LC-20AD nano-HPLC (Shimadzu, Kyoto, Japan) by the autosampler onto C18 trap column and the peptides were eluted to analytical C18 column (inner diameter 75 μm) packed in-house. Samples were then collected at a rate of 8 μL/min for 5 min. A 35 min gradient was run at 300 nL/min ranging from 8% to 35% of buffer B (2% H_2_O and 0.1% FA in ACN), followed by a 5 min linear gradient to 60% buffer B, maintenance at 80% of buffer B for 5 min and return to 5% on 0.1 min and equilibrated for 10 min. 

The peptide samples were then subjected to Nanospray III ionization of TripleTOF 5600 System, has a high mass accuracy, less than 2 ppm (SCIEX, Framingham, MA, USA). Data were acquired with the following MS conditions: ion spray voltage 2300 V, curtain gas of 30, nebulizer gas of 15, and interface heater temperature of 150 °C. The accumulation time for MS1 is 250 ms, and the mass ranges were from 350 to 1500 Da. Based on the intensity in MS1 survey as many as 30 product ion scans were collected if exceeding a threshold of 120 counts per second (counts/s) and with charge-state 2+ to 5+, dynamic exclusion was set for 1/2 of peak width (12 s). For iTRAQ data acquisition, the collision energy was adjusted to all precursor ions for collision-induced dissociation, and the Q2 transmission window for 100 Da was 100%.

### 4.7. Proteomic Data Analysis

Acquired raw MS/MS data were converted into MGF format by Proteo Wizard tool msConvert, and the exported MGF files were searched using Mascot version 2.3.02 against the selected database. The data attainment was performed with Analyst QS 2.0 software (Applied Biosystems/MDS SCIEX). Furthermore, the peptide and protein detection was performed through Mascot 2.3.02 (Matrix Science, London, UK), [62] against the NCBI for the *T*. *pseudonana C*CMP1335. For the protein identification mass tolerance of (0.05 Da) was permitted for intact peptide masses and (0.1 Da) for fragmented mass with an allowance of missed cleavage in the trypsin digests. Gln-pyro-Glu (N-term Q), oxidation (M), iTRAQ 8plex (Y) as variable modification, and carbamidomethyl (C), iTRAQ 8 plex (N-term), iTRAQ 8plex (K) as fixed modifications. 

The charge-states of peptides were set +2 to +5. Furthermore, an automated software IQuant, [63] used for analyzing labeled peptides with isobaric tags, which integrates Mascot Percolator, a well-performing machine learning method for rescoring database search results. To assess, the confidence level of peptide the PSMs was pre-filtered at a PSM-level FDR of 1%. Then based on the “simple principle” (the parsimony principle), identified peptide sequences are assembled into a set of confident proteins. For the reducing the probability of false identification a protein FDR at 1%, which is based on the picked-up protein FDR strategy, will also be estimated after protein inference (protein-level FDR ≤ 0.01). For the protein quantification, it was required that protein should contain a minimum of two unique peptides, and the protein ratios were weighted and normalized by the median ration in Mascot. Additionally, Mascot (2.3.02) was used to perform Student’s *t*-test. We only used ratio *p*-values < 0.05, and fold changes of >2 were considered as significant. 

### 4.8. Functional Annotation

The COG (Cluster of Orthologous Groups of proteins) and then GO (Gene Ontology) analyses (http://www.geneontology.org) were performed according to the method reported in the earlier literature [64]. The differentially regulated proteins in GO terms was carried out using the following formula:

$$P=1\;-\;\sum_{i=0}^{m\;-\;1}\frac{\left(\begin{array}{c} M\\ i \end{array}\right)\left(\begin{array}{c} N\;-\;M\\ n\;-\;i \end{array}\right)}{\left(\begin{array}{c} N\\ n \end{array}\right)}$$

where *N* is the number of all proteins with GO annotation information, *n* is the number of the differentially regulated proteins with GO annotation information, *M* is the number of proteins with a given GO term annotation, and *m* is the number of the differentially regulated proteins with a given GO term annotation. Furthermore, proteins with the two-fold change between each sample and a *p*-value less than 0.05 were determined as differentially expressed proteins to regulate cellular metabolism. The metabolic pathway analysis of differentially identified proteins was conducted according to the KEGG Pathway Database (http://www.genome.jp/kegg/), which represents knowledge in molecular interactions and reaction networks.

## 5. Conclusions

In this study, iTRAQ proteomic profile and physiological characteristics represent not only comprehensive systematic analysis of the diatoms intracellular silicate stress responses but also provides many previously uncharacterized cellular metabolism processes and proteins involved in the energy production, carbon metabolism, and ROS accumulation as shown in Figure 4 and Figure 6. The present finding demonstrates new molecular views of diatom response to silicate stress and their cell controlling mechanisms: (1) reduced electron flow in photosystem reduced the ATP production and controls the cell growth and replication, (2) reduction of reduced equivalents and carbon skeletons regulated central carbon metabolism and controlled carbon fixation in Si-limited cells, and (3) alteration of electron flow in the cellular process may lead to possible ROS accumulation. This proteomic study significantly facilitates our understanding of the intracellular processes of diatoms in response to silicate stress in the marine environments which could be valuable for future physiological research in diatoms.

## Supplementary Materials

Supplementary materials can be found at https://www.mdpi.com/1422-0067/20/10/2540/s1.

## Abbreviations

iTRAQ	Isobaric tag for relative and absolute quantification
ROS	Reactive Oxygen Species
ATP	Adenosine triphosphate
PSII	Photosystem II
PSI	Photosystem I
PCD	Programmed Cell Death
LC-MS/MS	liquid chromatography-tandem mass spectrometry
NPP	Net Primary Productivity
GO	Gene Ontology
COG	Clusters of Orthologous Groups
KEGG	The Kyoto Encyclopedia of Genes and Genomes
DEPs	Diffrenccilaly Expressed Proteins
FCPs	Fucoxanthin-chlorophyll proteins
GTP	Guanosine triphosphate
FADH2	flavin adenine dinucleotide
TCA	The citric acid cycle
NADH	Nicotinamide adenine dinucleotide
RuBisCO	Ribulose-1,5-bisphosphate carboxylase/oxygenase
