# Supplementary material for: Quantitative Proteomic Analysis Reveals Novel Insights into Intracellular Silicate Stress-Responsive Mechanisms in the Diatom Skeletonema dohrnii
Source: Int J Mol Sci. 2019 May 23;20(10):2540. doi: 10.3390/ijms20102540 (PMC6566588; doi:10.3390/ijms20102540)

Title: Quantitative Proteomic Analysis Reveals Novel Insights into Intracellular Silicate Stress-Responsive Mechanisms in the Diatom *Skeletonema dohrnii*

Authors: Thangaraj Satheeswaran, Shang Xiaomei, Sun Jun*, Liu Haijiao

Figure S1. An iPath metabolic biochemistry map of protein expressed and identified as down-regulated in the Si-limited and Si-added cells of *S. dohrnii*

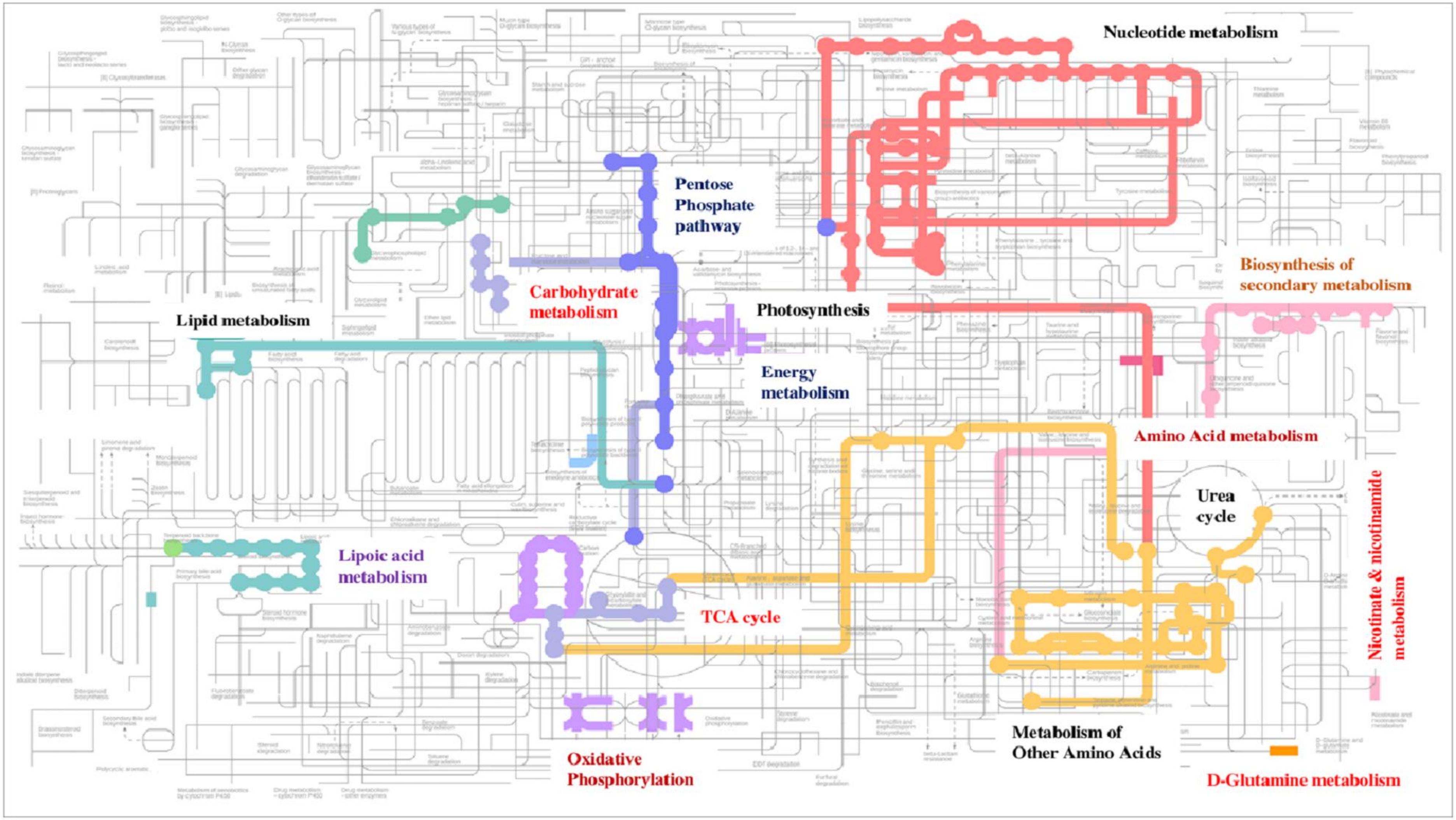

Supplement: Supplementary file 1 [file ijms-20-02540-s001.zip › IJMS-Figure S1.pdf]
